# Robotic Assisted Upper Limb Training Post Stroke: A Randomized Control Trial Using Combinatory Approach Toward Reducing Workforce Demands

**DOI:** 10.3389/fneur.2021.622014

**Published:** 2021-06-02

**Authors:** Aamani Budhota, Karen S. G. Chua, Asif Hussain, Simone Kager, Adèle Cherpin, Sara Contu, Deshmukh Vishwanath, Christopher W. K. Kuah, Chwee Yin Ng, Lester H. L. Yam, Yong Joo Loh, Deshan Kumar Rajeswaran, Liming Xiang, Etienne Burdet, Domenico Campolo

**Affiliations:** ^1^Interdisciplinary Graduate School, Nanyang Technological University, Singapore, Singapore; ^2^Robotic Research Center, Mechanical and Aerospace Engineering, Nanyang Technological University, Singapore, Singapore; ^3^Centre for Advanced Rehabilitation Therapeutics, Tan Tock Seng Hospital Rehabilitation Centre, Singapore, Singapore; ^4^NUS Graduate School for Integrative Sciences and Engineering, National University of Singapore, Singapore, Singapore; ^5^School of Physical and Mathematical Sciences, Nanyang Technological University, Singapore, Singapore; ^6^Department of Bioengineering, Imperial College of Science, Technology and Medicine, London, United Kingdom

**Keywords:** robotic rehabilitation, assessment, upper limb, randomized control trial, stroke

## Abstract

Post stroke upper limb rehabilitation is a challenging problem with poor outcomes as 40% of survivors have functionally useless upper limbs. Robot-aided therapy (RAT) is a potential method to alleviate the effort of intensive, task-specific, repetitive upper limb exercises for both patients and therapists. The present study aims to investigate how a time matched combinatory training scheme that incorporates conventional and RAT, using H-Man, compares with conventional training toward reducing workforce demands. In a randomized control trial (NCT02188628, www.clinicaltrials.gov), 44 subacute to chronic stroke survivors with first-ever clinical stroke and predominant arm motor function deficits were recruited and randomized into two groups of 22 subjects: Robotic Therapy (RT) and Conventional Therapy (CT). Both groups received 18 sessions of 90 min; three sessions per week over 6 weeks. In each session, participants of the CT group received 90 min of 1:1 therapist-supervised conventional therapy while participants of the RT group underwent combinatory training which consisted of 60 min of minimally-supervised H-Man therapy followed by 30 min of conventional therapy. The clinical outcomes [Fugl-Meyer (FMA), Action Research Arm Test and, Grip Strength] and the quantitative measures (smoothness, time efficiency, and task error, derived from two robotic assessment tasks) were independently evaluated prior to therapy intervention (week 0), at mid-training (week 3), at the end of training (week 6), and post therapy (week 12 and 24). Significant differences within group were observed at the end of training for all clinical scales compared with baseline [mean and standard deviation of FMA score changes between baseline and week 6; RT: Δ4.41 (3.46) and CT: Δ3.0 (4.0); *p* < 0.01]. FMA gains were retained 18 weeks post-training [week 24; RT: Δ5.38 (4.67) and week 24 CT: Δ4.50 (5.35); *p* < 0.01]. The RT group clinical scores improved similarly when compared to CT group with no significant inter-group at all time points although the conventional therapy time was reduced to one third in RT group. There were no training-related adverse side effects. In conclusion, time matched combinatory training incorporating H-Man RAT produced similar outcomes compared to conventional therapy alone. Hence, this study supports a combinatory approach to improve motor function in post-stroke arm paresis.

**Clinical Trial Registration:** www.ClinicalTrials.gov, identifier: NCT02188628.

## 1. Introduction

There is a growing number of patients who are suffering from sensorimotor disabilities (1), in part due to the aging population. In particular, stroke remains a leading cause of death and disability globally. Findings from the Global Burden of Disease Study of 2017 (2, 3) indicate that stroke was the third leading cause of mortality (accounting for over 6.1 million deaths) and one of five leading causes of morbidity worldwide (accounting for 132 millions disability-adjusted life-years). Hemiparetic weakness is common after stroke (4), affecting over 70% of stroke survivors and demands the need for intensive training as upper limb impairments remain in 40% of survivors (5, 6). Therefore, there is a pressing need for physical therapy and insufficient therapists to meet the high demand for intensive upper limb therapy. Hence, robotic devices are introduced as a potential solution to provide quality ensured upper limb intensive therapy and decrease therapists' workload (7–10).

Different approaches were developed to compare the effects of upper limb robotic therapy in contrast to conventional interventions for motor function recovery in stroke patients [for reviews, e.g., (11–13)]. The three main approaches available in literature are: (i) the addition of robotic therapy to conventional therapy (14–18), (ii) the full substitution of regular therapy with robotic therapy (19–30), and (iii) the use of combinatory training schemes that integrate conventional and robotic therapy (31–41).

To investigate the effects of additional robotic training (i), the therapy outcomes of two groups of stroke participants who underwent either only conventional therapy or conventional plus robotic therapy were compared. For instance, Yoo et al. (16) showed that the improvements in motor scales were significant in both of the conventional and the added robotic therapy groups and that the changes in clinical scales compared to baseline were significantly higher in the participants who additionally trained with the robot. This indicates that robotic devices can supplement conventional therapy to provide extra training. Instead of enhancing the standard care by providing additional training with robots, another approach consists of fully substituting conventional therapy with robotic assisted therapy (ii). This is commonly done by comparing robotic assisted therapy with dose matched (same training frequency and duration) and/or non dose matched regular therapy. For example, Rodgers et al. (29) recently conducted a study with 770 subjects in which they compared robotic assisted training with a dose matched enhanced upper limb therapy and a non dose matched usual care. The post training motor abilities of the stroke patients who trained with the robot were comparable to the ones who received enhanced or usual care. However, participants of the robotic assisted training group presented worse activity of daily living (ADL) performances. Other studies have investigated this approach (19–28); however, they have not studied whether the improvements in motor scales were retained after therapy. Thus, there is little evidence to support the full replacement of conventional therapy with robotic therapy. The last approach uses a dose matched combinatory training scheme (iii) to share the workload between the robot and therapists. For instance, Lee et al. (37) compared conventional therapy with a dose matched combination of conventional and robotic assisted therapy. Participants of the robotic group trained half of the total training time with the robot and the other half with therapists. Significant improvements in functional scales and reduced spasticity were observed in both groups, with no significant difference between the groups. Other studies (31, 34) with the same portion of therapy time spent with the robot found comparable changes in motor functions and improvements in ADL in both groups. However, there was no mention whether the motor and functional improvements were retained post therapy and only a few studies investigated if the motor improvements were translated into higher performances in ADL. This indicates that combinatory training schemes need to be further investigated as there is still limited clinical evidence that they can produce similar outcomes compared to conventional therapy.

These approaches showed promising results for the use of robotics in neurorehabilitation therapy. Nonetheless, fully substituting conventional therapy with robotic assisted therapy can deprive patients of interactions with therapists, which have been shown to modify the patient's pain experience during the therapy (42, 43). Robotic therapy can also be less personalized compared to conventional therapy and can be limited by the degree of freedom of the device. Moreover, additional robotic therapy has benefits of extra training (14, 16) but this comes at a greater financial cost. In contrast to the other approaches, the use of combinatory therapy showed promising results, allowing therapists to share the work with the robots (31, 34, 37, 39) while maintaining similar outcomes compared to conventional therapy alone. This approach offers the advantages of decreasing the therapists' workload, which can lead to reduced cost (34) and enhancement of their productivity. However, there is a need to further investigate the effects of this approach on ADL performances and whether the effects of robot aided therapy are retained after the training. In this study, we further examine the employment of a combinatory training instead of trying to replace standard approaches of therapy or to get better performance.

We aim to validate the clinical efficacy and the safety of a time matched combinatory training approach that integrates robotic assisted training using H-Man and 1:1 conventional therapy. Previous studies investigated combinatory training schemes where the time spent on 1:1 therapy was reduced to half of the total therapy time (31, 34, 37). In this study, the proportion of time spent on 1:1 conventional therapy was one third of the total therapy time and we examine if this combinatory approach, with two thirds substitution using an arm robot, compares with conventional therapy alone. In addition, we explore if the changes in motor functions are translated into improvements in functional abilities and if the training effects are retained after the therapy, as it is normally observed for conventional therapy.

In this study, we compared 1:1 conventional therapy with a time matched combinatory training scheme in a randomized clinical trial with 44 stroke subjects. The participants who underwent the combinatory training scheme spent one third of the total therapy time in a 1:1 physical training with a therapist. For the rest of the training, the participants used the robot H-Man, a two-degree of freedom and compact upper limb rehabilitation and assessment device (44–54), under the minimal supervision of a therapist. One major aspect of this training scheme is that both of the groups received the same amount of training sessions, at a similar frequency and for the same amount of time, i.e., three therapy sessions per week over a span of 6 weeks.

To study the therapy outcomes of this time matched combinatory training scheme compared to conventional therapy, we studied the evolution of the clinical scales until 18 weeks post training. The primary outcome was motor impairment assessed by the gain in Fugl-Meyer Motor Assessment Scale (FMA) at the end of training (week 6) compared to baseline. Quantitative measures derived from the robot were also examined as clinical scales are ordinal and therapist dependent and robotic metrics have shown to provide further insights into the motor recovery process (49). Finally, we explored the relationship between quantitative and clinical measures to investigate the potential of robotic metrics to predict clinical measures.

## 2. Methods

### 2.1. Study Design

A prospective, single-center, non-inferiority, outpatient randomized controlled trial with equal (1:1) allocation to two arm treatments was conducted over two years from 1st April 2016 to 31st April 2018. The study was conducted at the outpatient clinic of the Tan Tock Seng Hospital, Centre for Advanced Rehabilitation Therapeutics (TTSH-CART), Singapore, a tertiary rehabilitation center with direct links to a national stroke center.

Prior to screening, recruitment or research interventions, ethical approvals were obtained from National Healthcare Group Domain Specific Review Boards (DSRB 2014/00122). The study's protocol was registered under the National Clinical Trials Registry on 11th July 2014 (clinical trial ID: NCT02188628, www.clinicaltrials.gov).

### 2.2. Participants

Participants were consecutively identified through an inpatient stroke rehabilitation standing database and their involvement lasted a total of 24 weeks. Majority of subjects had completed inpatient rehabilitation at the centre's rehabilitation hospital. All subjects gave written informed consent prior to recruitment, randomization, and research interventions. The study was reviewed and approved by the National Healthcare Group Domain Specific Review Boards (NHG-DSRB 2014/00122).

Inclusion criteria for this study were: a first-ever stroke diagnosed by stroke neurologists or neurosurgeons and brain imaging, age between 21 and 85 years, time since stroke within 3–24 months, predominant arm motor function deficits with baseline FMA score between 20 and 50 or presence of motor ataxia, and the ability to understand instructions and give informed consent. Exclusion criteria for this study were: uncontrolled medical illnesses, pregnancy, life expectancy <6 months, inability to sit upright with support for <90 min due to postural hypotension or pressure intolerance, arm related contraindications to robot aided therapy such as shoulder pain [Visual Analog Scale (55), VAS > 4/10], spasticity [Modified Ashworth Scale (56), MAS > 2], severe sensory and visual impairments, hemi spatial neglect assessed using the line bisection test, and screening Mini-Mental State Examination score, MMSE <27/30.

**Figure 1:**
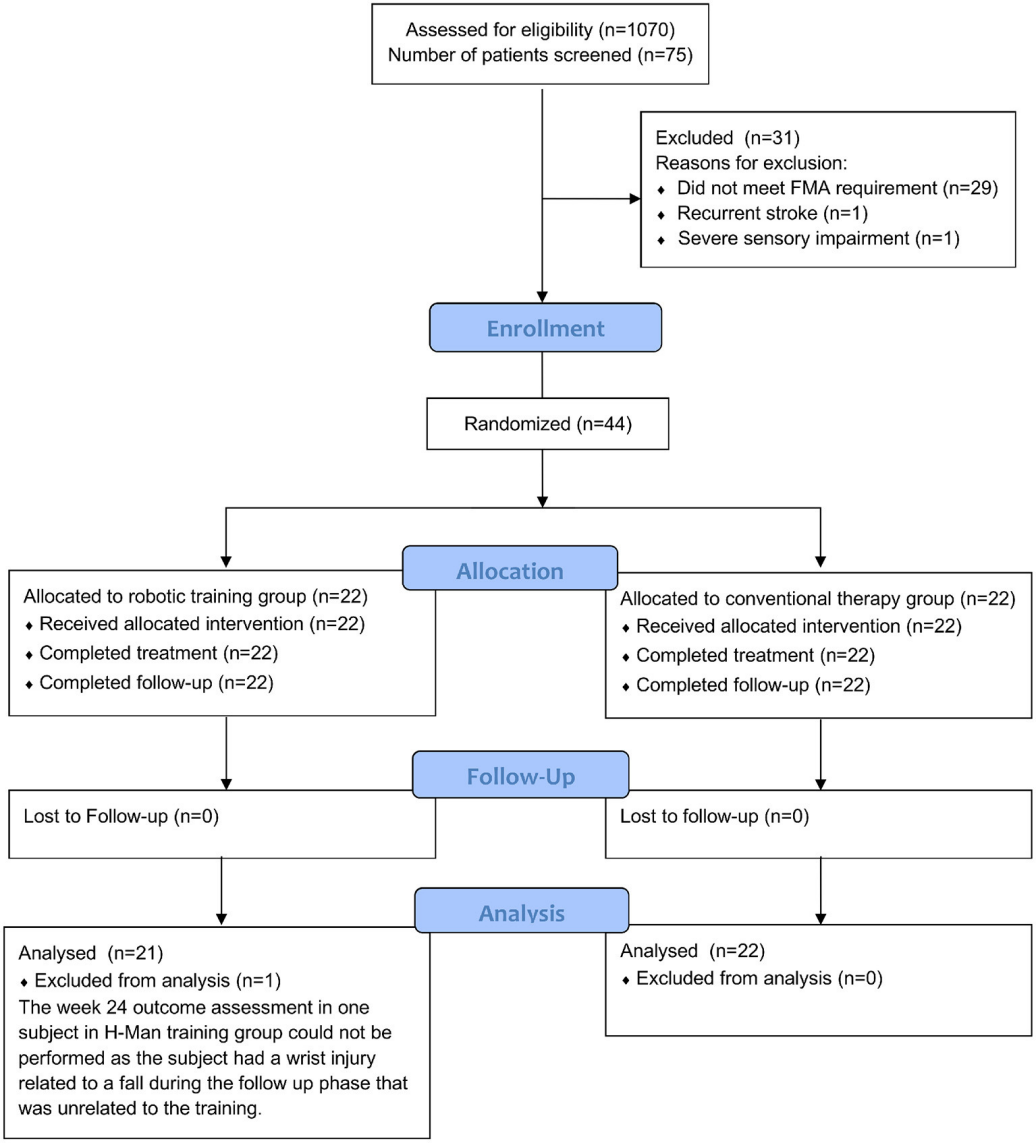
CONSORT diagram.

Figure 1 shows the subject recruitment and randomization process. In all, a total of 44 subjects were enrolled and randomized after screening 75 subjects.

### 2.3. Apparatus and User Interactions

#### 2.3.1. Apparatus

**Figure 2:**
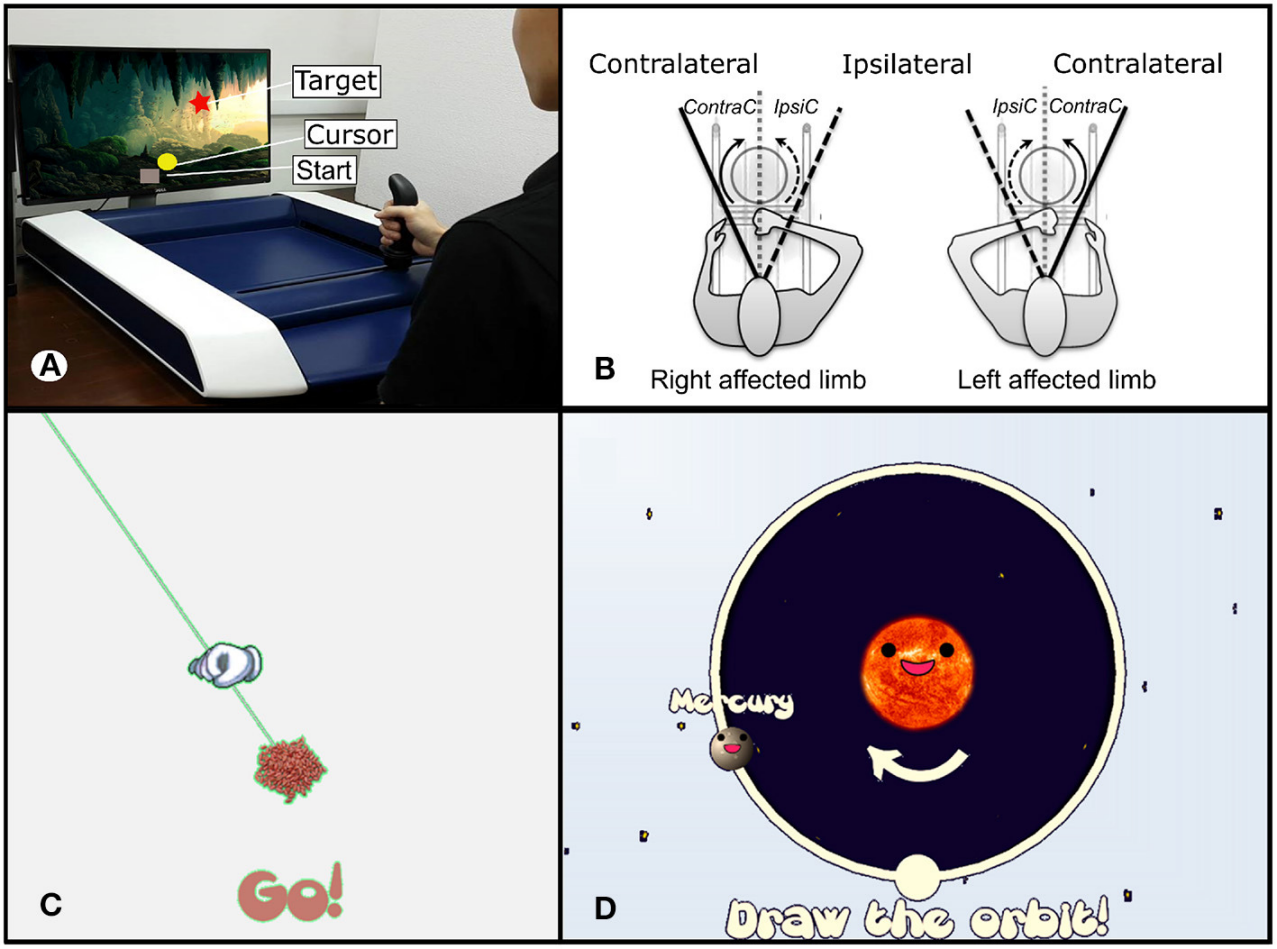
**(A)** Subject using the latest version of H-Man, a novel and compact upper limb rehabilitation and assessment robot. **(B)** Representation of the assessment task protocols for subjects using the left and the right hand, with direction notations. **(C)** Representation of the visual stimuli used for the line tracing task. **(D)** Visual stimuli of the circle tracing task.

The experimental apparatus used in this study was H-Man (Figure 2A), a two degree of freedom and compact robot designed by Campolo and colleagues at Nanyang Technological University, Singapore (44). It is characterized by a simple mechanical design and an H-shape cabled differential transmission. The unique features of H-Man are its intrinsic safety, its ease of control, owing to its homogenous workspace, and the fact that it is low-cost, compact and lightweight (about 7 kg). It was employed in post-stroke neurorehabilitation therapy, assessment of sensorimotor functions (49, 51, 53), and for human motor control experiments with healthy participants (45, 46, 48, 54).

#### 2.3.2. User Interactions

In the outpatient clinic, the participant was seated on a chair with a back support and the robot was situated in front of the subject on a fixed table. A flat screen monitor (32 inches) was placed in front of H-Man and delivered visual feedback throughout the experiment (Figure 2A). The visual stimuli developed in the UNITY software consisted of the task feedback and instructions. The starting posture was adjusted with respect to the subject such that the sternum was aligned with the handle of the robot, and the elbow bent at 90°. The initial position of the handle of the robot was set at approximately 25 cm from the sternum. Physical trunk restraint was used during training to limit trunk movements during the task executions. For subjects with severe distal weakness or impaired ability to grasp the handle of the robot, additional wrist and hand straps were provided.

### 2.4. Randomization and Blinding

Subjects were randomized with 1:1 allocation using computer-generated block of four numbered randomization codes into either H-Man robot training group or conventional therapy group, which was generated by Tan Tock Seng Hospital clinical research unit who were not involved in screening nor enrolment of subjects, and assignments were performed by the study team upon enrolment of subjects. Subjects were not blinded to group allocation, but occupational therapists who performed the clinical outcome assessments were blinded to the subjects' group allocation.

### 2.5. Training

The 44 stroke subjects were divided into two groups of 22 subjects to conventional and robotic therapy groups. Both of the groups received the same number of training sessions (*n* = 18) of 90 min each, three times a week and over a span of 6 weeks. The Conventional Therapy (CT) group received 90 min of 1:1 conventional therapy from a trained occupational therapist. The Robotic Therapy (RT) group underwent a 60 min robotic therapy session, minimally supervised by occupational therapists and bio-engineers, followed by a 30 min 1:1 conventional therapy session. During the robotic therapy, the subjects performed a point-to-point reaching task (in different shape patterns) with H-Man, which incorporated a performance based adaptive controller. The controller adjusts the interaction dynamics trial-by-trial based on an online estimation of patients task performance during a point to point reaching task, ranging from performance enhancement to performance degradation. The conventional therapy included passive mobilization and active-assisted approaches based on neuro-developmental techniques to enhance normal movement patterns, repetitive tasks, specific training for functional reach training (57, 58) and the use of upper limb inclined board and motorized arm bike. The study concluded when all recruited subjects completed training interventions and follow-up.

### 2.6. Assessment

#### 2.6.1. Protocol

The motor functions of the participants from both groups were assessed a total of five times over a period of 6 months: at baseline (week 0), mid-training (week 3), post training (week 6), and at two follow-ups (week 12 and 24). All participants received the same clinical and robotic assessments. Clinical outcome measures included the FMA (59), the Action Research Arm Test (ARAT) (60), and the Grip Strength (GS—Kilograms of Force: KgF) assessed via a digital dynamometer. Adverse events such as pain (Visual Analog Scale [0–100]), arm spasticity (Modified Ashworth Scale [0–4]), and study drop outs were also measured. Participants of the RT group reported outcome measures on their experience, satisfaction and perceived benefits of robotic assisted therapy using H-Man after 6 weeks of training interventions. All clinical assessments were performed by a qualified occupational therapist not involved in the training of subjects. In the robotic assessment, the subjects performed two assessment tasks with H-Man, i.e., line tracing (61) and circle tracing tasks (62), which were inherently different from the training task (point-to-point reaching) and these are described here.

#### 2.6.2. Robotic Assessment Tasks

Subjects were asked to perform the assessment tasks using both paretic and non-paretic arms, performing voluntary movements at their own speed following the instructions displayed on the screen. Prior to this, a trial session that lasted for few trials was conducted to familiarize the subject with the task.

##### 2.6.2.1. Line Tracing Assessment (LTA)

Participants were asked to move along a straight line and reach as far as possible within their range of motion, without any time constraint. The straight line was pseudo-randomly chosen among three directions (i.e., −45, 0, and +45° from the vertical axis) and was displayed on the screen during the whole duration of each trial (Figure 2C). At the beginning of each trial, the target movement direction was presented, and participants were asked to initiate their outward reaching movement. Participants were instructed to reach their maximum range of motion and then hold on to that position for three seconds (outward reaching movement). This was followed by an instruction to go back to the starting position (inward reaching movement). Six trials were carried out in each of the three directions (i.e., 18 trials in total).

##### 2.6.2.2. Circle Tracing Assessment (CTA)

This task was used to assess coordination. Given that hemiparesis could affect either arm, the independent variable movement direction was coded with respect to an egocentric frame of reference in the LTA (49) (Figure 2B). Similarly, for the CTA, the movement directions of the participants performing the task with the left hand were mirrored so that they were comparable across subjects. Participants were asked to trace a circle with a radius of 20 cm both in Ipsi Circular (IpsiC) and Contra Circular (ContraC) directions indicated by the visual feedback (Figure 2D). There was also no time constraint to complete the task. The trial ended when the participant had returned to the initial position. Subjects repeated the task six times in both directions in randomized order (i.e., 12 trials in total). A trial was considered successful if the subject could cover at least half the radius (10 cm) of the circle.

To maintain consistency, we refer to IpsiC as movement direction initialized toward the Ipsilateral direction and ContraC as movement initialized toward the contralateral direction (Figure 2B).

### 2.7. Data Analysis

The raw kinematic data (position) of the paretic arm's performance in both assessment tasks was filtered using a low pass filter (Butterworth: 6th order, cut-off frequency Fc: 20 Hz, sampling rate, Fs: 1,000 Hz). The filtered data were used in an offline data process to calculate multiple task performance indices adapted from literature. Out of these performance indices, few metrics describing the smoothness, temporal efficiency and task deviation error were identified based on reliability and sensitivity analyses similar to the procedure in (49, 51).

- ***Spectral ARC (SPARC):***The smoothness of each motion was assessed using the SPARC (Spectral ARC) metric (63). SPARC is a dimensionless, consistent, sensitive and robust metric validated to assess smoothness that is independent of temporal movement scaling. High values of SPARC correspond to high smoothness performance.
  (1) $$\begin{array}{r} SPARC=-\int_{0}^{w_{c}}\sqrt{(\frac{1}{w_{c}}{)}^{2}+(\frac{d\hat{V}\left(w\right)}{dw}{)}^{2}}dw \end{array}$$
  where $\hat{V}(w)$ is the normalized Fourier magnitude spectrum, normalized with respect to the DC magnitude *V(0)* and ω_*c*_, an adaptable cut off frequency, is calculated as
  (2) $$\begin{array}{r} \omega_{c}≜\min\left\{\omega_{c}^{max},\;\min\left\{\omega ,\hat{V}\left(r\right)<\bar{V}\;\forall \;r>\omega \right\}\right\} \end{array}$$
- ***Normalized Time to Peak Velocity:***The temporal efficiency is the ability to perform a task with a symmetrical and bell shaped velocity profile (64, 65). As the current tasks had no temporal constraints, the time to peak velocity normalized with the total movement time was considered as a suitable metric. The value lies in the range 0–1. In an ideal case, the time to peak velocity (*T*_*peak*_) occurs in the middle of the trial (50% of the movement time, *T*_*peakN*_ = 0.5) (65, 66). For further comparison across subjects with high variability and to test the nature of symmetry of velocity profiles, we removed the bias of 0.5 and took the absolute value. Any deviation thereof was estimated as a disability in temporal efficiency and is calculated by:
  (3) $$\begin{array}{r} T_{peakN}=\frac{T\left(V_{peak}\right)-T(V_{onset})}{T_{offset}-T_{onset}} \end{array}$$
  Time to Peak Velocity Normalized Absolute:
  (4) $$\begin{array}{r} T_{peakN\text{\_}Abs}=|T_{peakN}-0.5| \end{array}$$
  *T*(*V*_*peak*_) stands for the time when the maximum velocity is reached and *T*(*V*_*onset*_) is the time when 10% of the maximum velocity is reached. Here, the lower the value of *T*_*peakN*_*Abs*_, the higher the movement symmetry.
- ***Length of Curve Ratio (LOC):***The deviation from the desired path was considered as the task error measured by the length of curve ratio metric. A curve (a line for the LTA and an ellipse for CTA) was fitted on the subject's trajectory. The ratio of length of this fitted curve to the original trajectory curve gave the task deviation error. This is also called hand-path ratio and had been used as an assessment metric for task deviation in (67–69). In the ideal case, where there was no deviation from the desired path, the length of the curve ratio would be 1, so to remove any bias toward overshooting and undershooting and have a unidirectional measures, we removed the bias of 1 and calculated the LOC as:
  (5) $$\begin{array}{r} LOC=\left|\frac{y}{\hat{y}}-1\right| \end{array}$$
  where y and ŷ denote the observed length and the fitted curves, respectively. Here, the lower the LOC values, the smaller the task error.

For both tasks, only paretic arm assessment results are presented. For the LTA, the movement was divided into outward and inward movement segments based on the maximum distance covered and the peak velocity (49). In this paper, only the outward movement performance is analyzed and discussed.

### 2.8. Statistical Analysis

Use of modified intention to treat analyses was employed for the clinical datasets. Based on previous studies on robotic therapies for rehabilitation of stroke patients, a common standard deviation of the two groups is obtained as 37% of the baseline mean FMA score. With 80% power, the minimum sample size is found within the range of 14–19 for each group in a one-sided independent two-sample *t*-test at 5% level of significance. Given that a total of 40 stroke patients planned in the randomized controlled trial (20 subjects per arm), the power analysis for outcome FMA score guarantees a sufficient power between 82 and 91% for the aforementioned one-sided *t*-test. Considering a potential 10% drop out rate, the sample size needed would be 22 subjects per arm, a total of 44 subjects in the clinical trial.

For this study, both LTA and CTA had to be initiated by the subjects, hence some severely impaired subjects could not perform or initiate LTA and CTA tasks due to motor weakness or limb incoordination. Thus, a total of 34 complete robotic metric data sets at each time points were available for analysis: 15 in the CT group and 19 in the RT group. For each session, the median value across the trials of one of the directions for each robotic assessment (LTA: Ipsilateral; CTA: IpsiC) was taken as the representative performance of each subject.

#### 2.8.1. Clinical Outcomes and Quantitative Robotic Measures

In this study, we examined the effects of a time matched combinatory training scheme compared to a 1:1 conventional therapy on motor function recovery. First, we studied the evolution of both clinical and quantitative measures within each group to detect if the participants' motor functions significantly improved with therapy. Furthermore, we investigated potential differences in the effects of both training schemes by comparing the gains from baseline in clinical and quantitative measures of both groups. Non-parametric tests were used as data were not normally distributed (Shapiro-Wilk test) and the statistical significance level was set at 5% for all statistical tests. First, Wilcoxon Rank Sum Tests were performed to verify that there was no significant difference between the clinical outcomes and the robotic measurements of the RT and CT groups at baseline to ensure unbiased division of subjects. To explore the evolution of the clinical scores and robotic measurements over time within each group, Wilcoxon Signed Rank Tests were performed between the baseline assessment and the subsequent assessment sessions (mid-training, end of training and the two follow-ups) separately in the two groups. Wilcoxon Signed Rank Tests were used between the end of training session (week 6) and the last follow-up assessment session (week 24) within each group to assess whether the therapy outcomes were retained after training. To compare the effects of the two training between the groups, Wilcoxon Rank Sum Tests were run between the assessment measure changes compared to the baseline in each session of the RT and the CT groups.

The statistical analyses were done using Mathworks Matlab R2017b and IBM SPSS Version 25.

#### 2.8.2. Correlations Between Clinical and Quantitative Measures

Correlations between clinical and robotic assessment measurements were analyzed to investigate the potential of robotic measures to predict clinical assessment measures. The correlations were evaluated using Pearson correlation. All assessment sessions were combined for the correlations.

Because the metrics had different units, normalization was performed to scale the data such that each metric had 0 mean and a standard deviation of 1. Pearson correlation coefficients ranging from 0 to 0.39 were considered as weak, from 0.40 to 0.59 as moderate and from 0.60 to 1.0 as strong.

## 3. Results

### 3.1. Study

Figure 1 shows the flow diagram of the screened subjects throughout the study duration. In all, 75 eligible subjects were screened and 44 subjects who fulfilled all criteria were recruited and randomized into two groups of 22 subjects each per intervention arm. Altogether, 44 subjects (22 RT, 22 CT) completed training and 43 (21 RT, 22 CT) follow up clinical datasets were available for analyses.

For this sample of 44 subjects, their mean age was 55.46 (years), 25 were male (56.8%), and 19 were female (43.2%) while 22 had ischemic strokes (50%). The median stroke duration was 433 days (IQR 398.5) and the baseline mean upper limb FMA was 38.0/66 (SD 10.6). In both groups, arm paresis was moderate to severe with mean FMA of 40 or less, while there was a nearly equal proportion of haemorrhagic vs. ischemic strokes. An equal number of dominant and non-dominant arms were trained in both groups (15 dominant and 7 non-dominant arms trained in each RT and CT training groups). For the robotic assessment analyses, 34 complete datasets were analyzed (RT: 19 and CT: 15). The baseline demographic and clinical characteristics of the two treatment groups are provided in Table 1. No significant difference between the groups was observed at baseline in all robotic and clinical measures (*p*>0.05).

**Table 1:** Baseline demographic and clinical characteristics by training groups.

**Characteristics**	**Robotic therapy (RT)**	**Conventional therapy (CT)**
**Age—years**		
Mean (SD)	56.32 (10.37)	54.59 (10.92)
**Sex—No (%)**		
Male	11 (50)	14 (63.64)
Female	11 (50)	8 (36.36)
**Arm trained—No (%)**		
Dominant arm	15 (68.18)	15 (68.18)
Non-dominant arm	7 (31.82)	7 (31.82)
**Side of stroke—No (%)**		
Left	14 (63.64)	15 (68.18)
Right	8 (36.36)	7 (31.82)
**Post stroke duration—days**		
Median (IQR)	458 (451.3)	390 (327.5)
**Type of stroke—No (%)**		
Haemorrhagic	10 (45.45)	12 (54.55)
Ischemic	12 (54.55)	10 (45.45)
**Clinical outcomes—Mean (SD)**		
Fugl-meyer assessment (FMA)	40.23 (9.29)	35.86 (11.65)
Action research arm test (ARAT)	26.64 (16.64)	18.86 (15.63)
Grip strength (GS)	7.49 (3.22)	6.72 (4.12)

*No significant difference was observed in all of the above parameters between the RT and the CT groups (p>0.05). RT, robotic therapy; CT, conventional therapy; SD, standard deviation; IQR, interquartile range*.

The outcome measures related to the ease of use of H-Man, comfort, perceived benefit and satisfaction of H-Man training reported by the robotic therapy group subjects were recorded at week 6 i.e., the end of training (refer to Supplementary Figure 1). These were generally positive with 77% of the robotic group subjects were very or completely satisfied with the combinatory robotic therapy, 86% reported high scores with regard to ease of use, 91% agreed that the visuals used during the robotic therapy were easy to understand, 87% agreed that the robotic therapy set-up was comfortable; and finally, 86% of the subjects agreed that the training was useful for exercising their arms.

### 3.2. Study Adverse Events

In both intervention groups, there were no training-related adverse side effects or drop outs up to week 6 of the study (refer to CONSORT Diagram, Figure 1). In general, no increase in pain, spasticity or other adverse side effects were measured or reported by the participants (refer to Supplementary Figures 2–4). One subject in the H-Man intervention arm suffered an unrelated traumatic wrist injury during week 24 of the follow up period and was not able to perform week 24 assessments for both clinical and robotic outcomes. There were no other protocol deviations during the clinical trial.

### 3.3. Change in Clinical Outcome Measures Across Sessions

#### 3.3.1. Within Treatment Groups

**Figure 3:**
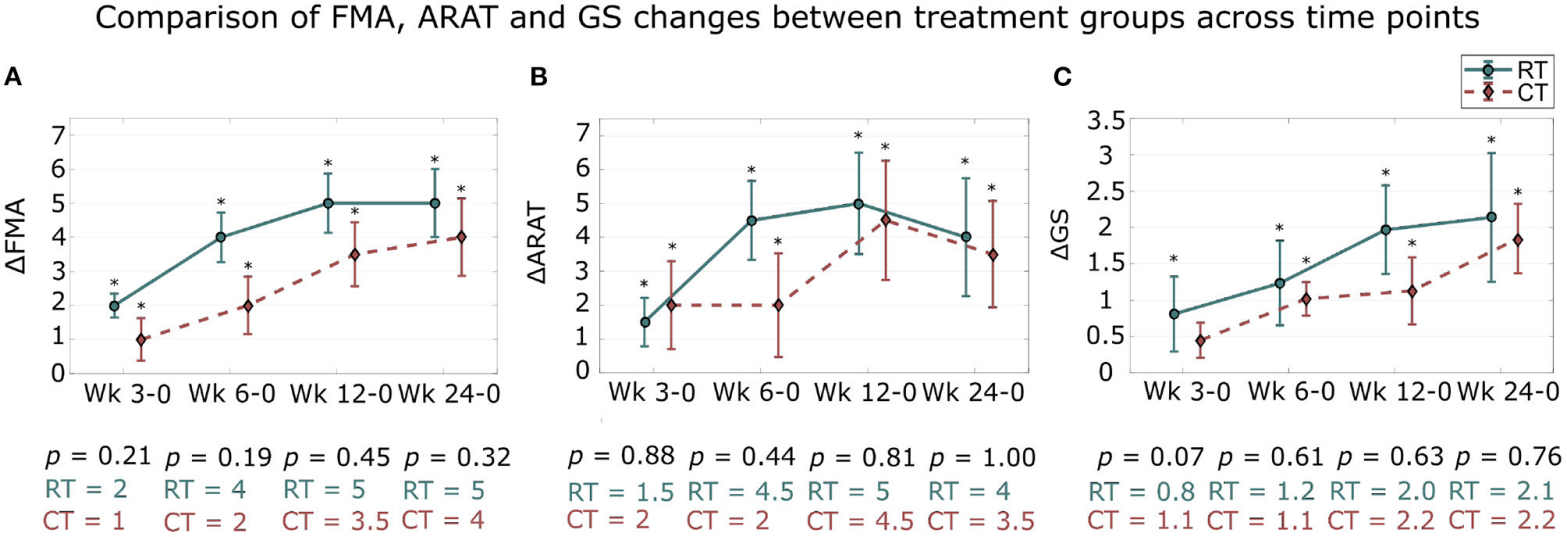
Changes in clinical outcome measures from baseline assessment across sessions. The changes in clinical measures over 24 weeks in comparison with the baseline assessment (week 0) are presented. Median and standard error of the changes in: **(A)** FMA, **(B)** ARAT, and **(C)** GS for the robotic therapy (RT) and conventional therapy (CT) groups are shown. The * signifies that the changes compared to baseline are significant (*p* < 0.05). Wk, week. The indicated *p*-value corresponds to the between treatment group differences in clinical scale changes compared to baseline at each assessment session.

Overall, significant improvements in all clinical assessment measures were observed in both RT and CT groups (Figure 3, Table 2).

**Table 2:** Clinical outcome measures by training group over time points.

	**Week 0**		**Week 3**		**Week 6**		**Week 12**		**Week 24**	
**Mean**	**RT**	**CT**	**RT**	**CT**	**RT**	**CT**	**RT**	**CT**	**RT**	**CT**
**(SD)**	**(*n* = 22)**	**(*n* = 22)**	**(*n* = 22)**	**(*n* = 22)**	**(*n* = 22)**	**(*n* = 22)**	**(*n* = 22)**	**(*n* = 22)**	**(*n* = 21)**	**(*n* = 22)**
FMA	40.23	35.86	42.5	37.73	44.64	38.86	45.05	40.27	45.33	40.36
	(9.30)	(11.65)	(9.34)	(11.93)	(9.77)	(11.69)	(10.35)	(11.95)	(11.43)	(11.57)
ARAT	26.64	18.86	28.95	22.18	31.91	23.77	32.5	24.95	31.57	24.59
	(16.64)	(15.63)	(17.11)	(17.18)	(17.73)	(18.31)	(19.10)	(18.38)	(19.69)	(16.94)
GS	7.49	6.72	8.77	7.14	9.41	7.81	9.98	8.41	10.86	8.94
	(3.22)	(4.12)	(4.87)	(3.83)	(4.84)	(3.70)	(4.92)	(4.08)	(6.28)	(4.01)

*SD, standard deviation; n, number of subjects; RT, robotic therapy; CT, conventional therapy; FMA, fugl-meyer motor assessment; ARAT, action research arm test; GS, grip strength*.

Statistical analysis showed significant improvements in FMA scale compared to baseline at all subsequent assessment sessions for the RT and the CT groups (both groups, all sessions: *p* < 0.05; Figure 3A). Moreover, the therapy benefits were retained between the end of training (week 6) and the last follow-up assessment sessions (week 24) for the RT group (*p* > 0.05), while the FMA scores continued to increase significantly after the end of the therapy in the CT group (between week 6 and 24; *p* = 0.04).

A similar trend was observed for the ARAT scale (Figure 3B). The gains in the ARAT scores were significant in all subsequent assessment sessions for the RT group, as well as for the CT group (both groups, all sessions: *p* < 0.05). Moreover, the changes in ARAT scores were retained post-treatment in both groups (*p* > 0.05).

Finally, significant improvements were observed in both groups over time for the GS (Figure 3C). Significant gains in GS compared to baseline were observed in the RT group at mid-training and at the end of training assessment sessions (week 3 and 6: *p* < 0.05) while there were also significant changes in GS in the CT group but only at the end of training session (week 3: *p* = 0.07; week 6: *p* < 0.05). Changes in the GS after therapy were significant in both groups and at both follow-up assessment sessions (week 12 and 24; both groups: *p* < 0.05). Patients in both groups continued to improve significantly after training with significant differences in GS between the end of training and the last follow-up assessment sessions (both groups: *p* < 0.05).

These results show that, similar to the CT group, the motor functions of the stroke participants of the RT group significantly improved over time and that the training effects were retained post treatment.

#### 3.3.2. Between Treatment Groups

Overall, there were no significant between group differences at all time points for FMA, ARAT, and GS clinical scales (Figure 3, Table 2).

While the subjects of the RT group had numerically larger FMA gains at the end of training and at the last follow up session (week 6 and 24) compared to the CT group (Week 6: RT: 4.41 and CT: 3.00; Week 24: RT: 5.38 and CT: 4.5), this did not reach statistical significance (Week 6: *p* = 0.19; Week 24: *p* = 0.32) (mean ± standard deviation of the changes compared to baseline; RT [points]: Wk 0–6: 4.41 ± 3.46; Wk 0–24: 5.38 ± 4.67 and CT [points]: Wk 0–6: 3.00 ± 4.00; Wk 0–24: 4.50 ± 5.35).

For the ARAT scale, the RT group showed numerically higher improvements compared to the CT group. Similarly to the FMA scale, there was no significant difference between the improvements of the RT and the CT groups at all assessment sessions (*p* > 0.05, all sessions) (RT [points]: Wk 0–6: 5.27 ± 5.45; Wk 0–24: 5.62 ± 8.15 and CT [points]: Wk 0–6: 4.91 ± 7.16; Wk 0–24: 5.73 ± 7.38).

Similarly, no significant difference between the changes in each group was found for GS (all sessions: *p* > 0.05) although the RT group tended toward higher improvements in GS compared to the CT group (RT [KgF]: Wk 0–6: 1.92 ± 2.73; Wk 0–24: 3.21 ± 4.15 and CT [KgF]: Wk 0–6: 1.08 ± 1.08; Wk 0–24: 2.22 ± 2.24).

### 3.4. Distribution of Quantitative Measures Across Sessions in the Line Tracing Task

**Figure 4:**
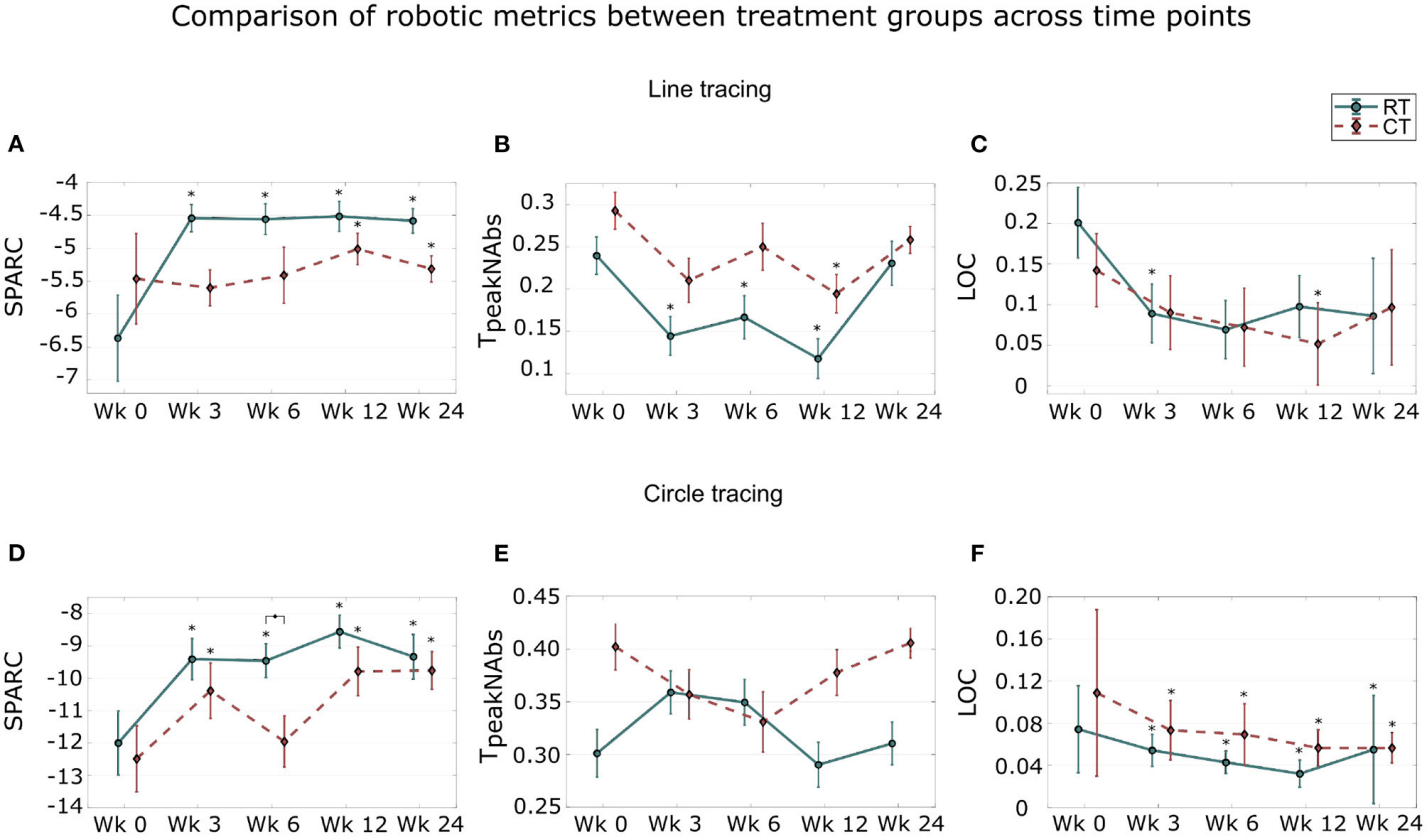
Distribution of quantitative measures across sessions in the line tracing assessment **(A–C)** and in the circle tracing assessment tasks **(D–F)**. Median and standard error of: smoothness, measured by SPARC; temporal efficiency, measured by normalized time to peak velocity (*T*_*peakN*_*Abs*_); and task deviation error, measured by Length of Curves (LOC) across the five assessment sessions are shown. The * signifies that the scores are significantly different from the baseline scores (*p* < 0.05). The ◇ signifies a significant difference in the changes of robotic measures between both groups (*p* < 0.05). Wk, week.

Overall, for the LTA, significant improvements were observed in all robotic metrics in both groups, with no significant differences between the groups (Figure 4, Table 3).

**Table 3:** Robotic metric outcome measures by training group over time points (*n* = 34).

	**Week 0**		**Week 3**		**Week 6**		**Week 12**		**Week 24**	
**Mean (SD)**	**RT**	**CT**	**RT**	**CT**	**RT**	**CT**	**RT**	**CT**	**RT**	**CT**
LTA–Smoothness	−7.03	−6.67	−4.83	−5.41	−4.76	−5.53	−4.90	−4.93	−4.76	−5.04
	(2.85)	(2.66)	(0.91)	(1.06)	(1.01)	(1.65)	(0.98)	(0.93)	(0.81)	(0.77)
LTA–Time	0.25	0.29	0.18	0.24	0.19	0.25	0.17	0.21	0.22	0.26
	(0.10)	(0.08)	(0.10)	(0.10)	(0.11)	(0.11)	(0.10)	(0.09)	(0.11)	(0.06)
LTA–Task error	0.22	0.20	0.16	0.17	0.15	0.14	0.18	0.15	0.18	0.18
	(0.19)	(0.17)	(0.16)	(0.18)	(0.16)	(0.19)	(0.17)	(0.20)	(0.31)	(0.28)
CTA–Smoothness	−13.03	−13.6	−10.2	−11.71	−9.40	−12.23	−9.20	−10.12	−9.59	−10.15
	(4.31)	(3.95)	(2.75)	(3.31)	(2.24)	(3.06)	(2.19)	(2.89)	(3.0)	(2.24)
CTA–Time	0.33	0.37	0.33	0.35	0.32	0.31	0.30	0.35	0.30	0.39
	(0.10)	(0.09)	(0.09)	(0.09)	(0.09)	(0.11)	(0.09)	(0.08)	(0.09)	(0.06)
CTA–Task error	0.15	0.22	0.07	0.12	0.06	0.11	0.06	0.08	0.10	0.08
	(0.18)	(0.31)	(0.07)	(0.11)	(0.05)	(0.11)	(0.06)	(0.07)	(0.22)	(0.06)

*SD, standard deviation; n, total number of subjects; RT, robotic therapy; CT, conventional therapy; LTA, line tracing assessment; CTA, circle tracing assessment; Smoothness, calculated by Spectral ARC (SPARC); Time, calculated by Normalized time to peak velocity; Task error, calculated by length of curve ratio*.

#### 3.4.1. Smoothness

The results for the LTA indicated significant improvements in smoothness compared to baseline during and after training in the RT group (Figure 4A; all sessions: *p* < 0.05). Compared to the RT group, no significant gains were found in the CT group during training (week 3 and 6: *p* > 0.05). The gains compared to baseline were significant post training in both groups (week 12 and 24, both groups: *p* < 0.05). Furthermore, comparisons between the end of training (week 6) and the last follow-up assessment sessions (week 24) revealed that the therapy effects were retained in both groups (both groups; *p*> 0.05).

When comparing across groups, the RT group tended to show higher improvements in smoothness during and after therapy compared to the CT group (Table 3). This was not further verified as there was no statistical difference in smoothness improvements between the groups at each assessment session (all sessions: *p* > 0.05; week 3: *p* = 0.12; week 6: *p* = 0.13).

#### 3.4.2. Temporal Efficiency

There were significant improvements in temporal efficiency during the therapy only for the RT group (week 3 and 6: *p* < 0.05) while there were significant improvements in both groups at the first follow-up assessment session (both groups, week 12: *p* < 0.05; Figure 4B). Comparisons between the end of training and the follow-up sessions showed retention of temporal efficiency gains in both groups (*p* > 0.05).

Comparing the two groups, the stroke participants from the RT group tended to perform better than the CT group in terms of improvement of symmetric velocity profile (Table 3). However, this was not translated into significant difference between the group with *p* > 0.05 in all sessions.

#### 3.4.3. Task Error

The decrease in task error was significant or marginally significant during training for the RT group (week 3: *p* = 0.02; week 6: *p* = 0.053) and post-training for the CT group (week 12: *p* = 0.04; week 24: *p* = 0.055, Figure 4C). The changes in task error were retained post-training (both groups; *p* > 0.05).

The improvements in task error performance were similar in the two groups as no significant difference was observed at each assessment session.

### 3.5. Distribution of Quantitative Measures Across Sessions in the Circle Tracing Task

For the CTA, significant improvements in smoothness and task error were observed. The improvements in smoothness performances of the RT group were significantly higher to the ones of the CT group at the end of the therapy.

#### 3.5.1. Smoothness

In the CTA, the RT group showed significant improvements in smoothness compared to baseline at all subsequent sessions (all session: *p* < 0.05; Figure 4D). The CT group also presented significant improvements at all subsequent sessions except for the end of training sessions (week 3, 12 and 24: *p* < 0.05, week 6: *p* > 0.05). Improvements in smoothness performances were retained post-training in the RT group (*p* > 0.05) while there were significant improvements between the end of training and the last follow-up sessions for the CT group (*p* < 0.05).

When comparing the two groups, the RT group showed higher improvements in smoothness at the end of the therapy compared to the CT group (Figure 4D). This was further verified as there was a significant difference between the RT and the CT groups at the end of training assessment sessions (week 6; *p* = 0.048).

#### 3.5.2. Temporal Efficiency

No significant difference between the baseline and every subsequent sessions was observed in both groups (all sessions and both groups; *p* > 0.05; Figure 4E). The therapy effects were retained with no significant difference between the post-training (week 6) and the last follow-up in the RT group (week 24); however, the therapy effects were not retained for the CT group (*p* = 0.03).

When comparing the two groups, there was no significant difference between the groups in temporal efficiency improvements during and after training (all sessions; *p* > 0.05).

#### 3.5.3. Task Error

Comparisons between the baseline assessment session with every subsequent sessions indicated that the decrease in task error was significant at all assessment sessions, in both groups (all session, both groups: *p* < 0.05; Figure 4F). The therapy effects were retained in the RT group (*p* > 0.05) while the task error was significantly smaller at the last follow-up compared to the end of training session for the CT group (*p* = 0.03).

When comparing across groups, there was no significant difference in the changes of task error between the groups at each assessment session (all sessions; *p* > 0.05).

### 3.6. Correlation Between Clinical and Robotic Measurements

In general, correlations between clinical scale changes and robotic measures in both robotic assessment tasks were found to be weak (Table 4). The greatest correlations were found between the task error of the CTA and ARAT (*r* = −0.35) and FMA scales (*r* = −0.34). For the LTA, the greatest correlation was found between the FMA scale and the smoothness outcomes (*r* = 0.31). These results indicate significant but weak correlations between robotic metrics and clinical scales.

**Table 4:** Results of the correlations between the clinical scales and the quantitative robotic measurements of both robotic assessment tasks (line tracing and circle tracing).

	**Line tracing**			**Circle tracing**		
	**Smoothness**	**Time**	**Task error**	**Smoothness**	**Time**	**Task error**
FMA	0.31*	−0.20*	−0.12	0.23*	0.03	−0.34*
ARAT	0.28*	−0.17*	−0.08	0.27*	0.01	−0.35*
GS	0.22*	−0.37*	−0.21*	0.12	−0.14	−0.21*

(Stroke participants, n = 34). Pearson correlation coefficient r. The * signifies p < 0.05. Smoothness, calculated by Spectral ARC (SPARC); Time, calculated by normalized time to peak velocity; Task error, calculated by length of curve ratio; FMA, Fugl-meyer motor assessment; ARAT, action research arm test; GS, grip strength.

## 4. Discussion

The aim of this study was to investigate the efficacy and the safety of a combinatory training scheme which integrates 1:1 conventional therapy (one third of total therapy time) and minimally supervised robotic therapy using H-Man (two thirds of total therapy time). In this study with 44 stroke patients, we compared the training effects of this combinatory training scheme with time matched conventional therapy alone. Overall, the combinatory training scheme using H-Man was safe, efficacious and acceptable in a supervised manner.

### 4.1. Similar Improvements With the Combinatory Training Scheme Compared to Conventional Therapy, an Approach to Reduce Therapists' Workload

The participants from both the conventional and the robotic treatment groups presented significant gains in all clinical scales (FMA, ARAT, and GS scales). Although the participants who underwent the combinatory training scheme only trained with an occupational therapist for one third of the total therapy time, they showed similar improvements in all clinical scales compared to the conventional therapy group. Moreover, the improvements in clinical scores were clinically relevant in the FMA scale for the robotic group. In fact, the minimal detectable change (MDC) is 5.2 points, as described in Wagner et al. (70), and the clinically important differences (CID) ranges between 4.25 and 7.25 points (71) for the FMA. At the end of training (week 6), only the robotic group achieved CID gains in FMA compared to the conventional group [4.41 (RT) vs. 3.0 (CT)]. Thus, the 6 weeks combinatory robotic training program resulted in numerically faster gains on average in FMA compared to 1:1 conventional therapy alone. In both groups, retention of motor gains was achieved, exceeding the CID 18 weeks after cessation of training. Moreover, FMA gains exceeding MDC were achieved at the end of the follow up period for RT group only. In ARAT, the gains from baseline were also clinically relevant as it exceeded the ARAT MDC of 5.7 (72) at the first follow-up for the RT group and at the first and last follow-up for the CT group.

Despite a relatively short training protocol, therapeutic gains were retained at least 18 weeks after the end of the training in both groups. In fact, the duration of the training was 6 weeks compared to other studies where the training duration varied between 8 and 12 weeks (21, 23, 26, 29). The continued gains in FMA and ARAT between the end of training and the last follow up session implies sufficient repetition practice for long term enduring gains. Thus, this combinatory approach is effective in achieving functionally meaningful gains in FMA and ARAT. Moreover, in the robotic group, the gains were achieved at a faster rate at the primary endpoint of training completion with the ability to sustain gains up to 18 weeks after training. This attests to the adequacy of practice intensity, repetition of reaching movements and type of training.

Contrary to recent studies (11, 21, 29), we observed that the results support the use of robot assisted training to improve stroke motor recovery using H-Man. Furthermore, previous studies (31, 34, 35, 37) indicated that the time spent in conventional therapy could be reduced by one half of the total therapy time with the use of combinatory robotic assisted training scheme. However, there was no evidence whether the therapy effects were retained after the training and if they were translated into functional improvements. In our study, we showed that the time spent on 1:1 conventional therapy could be reduced to one-third of the total therapy time using robotic aided therapy with H-Man, while providing similar outcomes compared to conventional therapy alone. We also showed that the gains in motor functions were translated into functional improvements, as supported by ARAT gains of 5.9 at week 12 for the RT group that reaches MDC, and that the therapy outcomes were retained at least 18 weeks following the end of training.

A potential clinical application of our study could include the partial substitution of conventional therapy with a safe, effective and feasible robotic aided therapy such as H-Man. This potentially can accelerate attainment of meaningful functional motor gains of stroke survivors. However, the efficacy of various proportions remains to be studied. Furthermore, instead of fully substituting conventional therapy in favor of robotic aided therapy, a potential vision for robotic therapy is to share the workload between the robot and the therapists. In fact, in our study, the robotic device automatically adapts according to the patients' performance, limiting the amount of interaction required during the training process. This will allow them to spend time in conventional therapy to translate motor gains to functionally useful and meaningful tasks for patients, without increased staff numbers. Hence, the combinatory training scheme presented in this study gives confidence that robotic aided therapy is an effective and safe therapeutic partner in the rehabilitation milieu and would potentially be able to enhance therapists' productivity via minimally supervised robotic aided therapy.

The therapists may also be able to refine their interventions to maximize the therapy outcomes. Since repetitive and intensive tasks can be handled by robotic devices, the therapists could focus on providing more individualized training and exercises that could not be administered by the robot alone. Thus, patients would benefit from intensive practice driven by robotic aided therapy and contextual real-world therapy by occupational therapists. Future studies could also explore the outcomes of other available treatments such as electromyographic biofeedback, sensorimotor training, and mental practice with motor imagery [reviewed in (73)] in combination with robotic assisted therapy.

Through integration of robotic aided therapy in clinical practice, therapists could manage several patients at the same time, reducing the workforce related therapy costs. Also, even though the full substitution of conventional therapy by robotic devices can be more expensive than regular therapy (29), some combinatory training schemes, where the training work is shared between the therapists and the robot, can be cheaper than conventional therapy alone (34, 74).

Apart from reducing hospital treatment costs, the use of robotic therapy in decentralized settings could also help to further reduce the therapy costs. For instance, instead of providing each training session with a mix of both conventional and robotic therapy, the therapy schedule could be, for example, designed as follow: first weeks of therapy with conventional therapy followed by robotic therapy for the rest of the therapy, as shown in (32). This could allow the patients to train in decentralized settings, or even at home (75, 76), which could reduce hospital treatments and transport related costs (77, 78).

### 4.2. Robotic Measures to Provide Additional Information on the Subject's Progress

In addition to conventional scales, we investigated the changes in quantitative metrics derived from the robotic assessment tasks. We observed that the subjects from both groups performed and retained smoother movements with better temporal efficiency and reduced task deviation errors compared to their baseline performances. Furthermore, these measures showed more sensitivity compared to the clinical scales as they detected a significant difference between the groups, unlike clinical scales. In fact, in the circle tracing task, the movement smoothness of the robotic therapy group improved significantly better compared to the conventional therapy group. This demonstrates the sensitivity of robotic task metrics to detect differences in movement kinematic performances. These results are consistent with the study (49) that showed the sensitivity of robotic metrics to track the performance changes of stroke patients.

Apart from giving sensitive insight on the patients' movement kinematic, robotic scales also provide the advantages of being sensitive, (semi-) autonomous, potentially more objective than clinical assessments, and less prone to human error/subjectivity (30, 79–81). However, their usability in assessing sensorimotor impairment in clinical settings is scarce. One of the major setbacks is the limited understanding of the relationship between robotic metrics and clinical scales. To determine if robotic measurements can be used to predict gold standard clinical outcome measures such as FMA, ARAT, and GS, we analyzed the correlations between the robotic measures and the clinical assessment outcomes. Overall, the results showed significant weak correlations. These results are consistent with the review of Tran et al. (82) who also found significant weak or moderate correlations among thirteen articles which studied the correlations between kinematics and clinical measures. This may indicate that the robotic metrics used in this study do not strongly relate to clinical scales but instead add further insights on the patient's progress. Hence, the results promote the use of robotic metrics derived from H-Man to provide additional and sensitive information to track the progress of stroke participants in an unobtrusive manner.

### 4.3. Study Limitations

This study has limiting factors to be considered. The minimum baseline FMA of participants to be included in the study was set to 20 points due to the nature of the robotic training. Furthermore, although the follow up was 6 months, it could be interesting to note a longer follow up period to study the long term gains after training. In terms of demographics of the sample, the mean age of the study population of 44 subjects was 55.5 years which is younger than the global stroke age which is around 60 years. Lastly, global measures of ADL competency, functional dependency and quality of life were not studied.

### 4.4. Future Work

We envision with this combinatory approach that several robots could be used with multiple patients simultaneously while being supervised by fewer therapists and such solutions could potentially be used in home settings. Future usability trials in community settings or nursing homes are needed to validate the versatility and access of such robot aided therapy for the majority of stroke survivors. Furthermore, it is important to ensure that robotic solutions remain affordable to achieve seamless deployment from acute hospitals to the community.

Beyond motor function and motion kinematic assessments, robotic devices could also be used to augment sensorimotor assessment by providing much needed proprioceptive assessment (50, 52), which is poorly represented on standardized clinical scales, to get better insight on the evolution of each individual patients. Lastly, future work could extend the application of this combinatory approach using H-Man to treat acute stroke.

## 5. Conclusion

Time matched combinatory robotic therapy that integrates robotic aided therapy and therapist supervised therapy in a 2:1 ratio, using H-Man, was safe, efficacious, and acceptable in a supervised manner.

The stroke participants who underwent this combinatory training showed comparable improvements in motor function and higher performances in movement smoothness compared to stroke participants who underwent conventional therapy alone for the same duration. The motor and functional improvements were also retained following the therapy. This promotes the use of the present robotic therapy program for 18 weeks to improve stroke motor recovery and reduce therapists' workload which can lead to reduced human effort and enhanced productivity.

## Data Availability Statement

The datasets presented in this study can be found in online repositories. The names of the repository/repositories and accession number(s) can be found at: https://github.com/Adelech/RCTData.

## Ethics Statement

The studies involving human participants were reviewed and approved by National Healthcare Group Domain Specific Review Boards (NHG-DSRB 2014/00122). The patients/participants provided their written informed consent to participate in this study.

## Author Contributions

The conception and the development of the study protocol were carried out by AH, AB, SC, SK, KC, and DC. The hardware and software setup were designed by AH, SC, SK, and AB. Clinical study and data from stroke patients were collected by AB, SC, SK, LY, DV, CK, and KC. Data analysis and statistics were performed by AC, AB, AH, SC, and KC. The IRB was prepared by DC, AB, AH, CK, and KC. The manuscript was prepared and written by AC, AB, AH, KC, EB, DC, SK, and CK. All the authors read and approved the final manuscript.

## Conflict of Interest

AH and DC hold equity positions in ARTICARES Pte. Ltd., a company that manufactures the type of technology used under license from Nanyang Technological University, Singapore. The remaining authors declare that the research was conducted in the absence of any commercial or financial relationships that could be construed as a potential conflict of interest.

## Abbreviations

RAT: robot-aided therapy
RT: robotic therapy
CT: conventional therapy
FMA: Fugl-Meyer motor assessment
ADL: activity of daily living
ARAT: action research arm test
GS: grip strength
LTA: line tracing assessment
CTA: circle tracing assessment
IpsiC: Ipsi circular
ContraC: contra circular
SPARC: spectral ARC
LOC: length of curve ratio
MDC: minimal detectable change
CID: clinically important difference.
