# Supplementary material for: Robotic Assisted Upper Limb Training Post Stroke: A Randomized Control Trial Using Combinatory Approach Toward Reducing Workforce Demands
Source: Front Neurol. 2021 Jun 2;12:622014. doi: 10.3389/fneur.2021.622014 (PMC8206540; doi:10.3389/fneur.2021.622014)
Supplement: Supplementary file 1 [file Data_Sheet_1.docx]

Supplementary Material

# Supplementary Figures and Tables

## Supplementary Figures


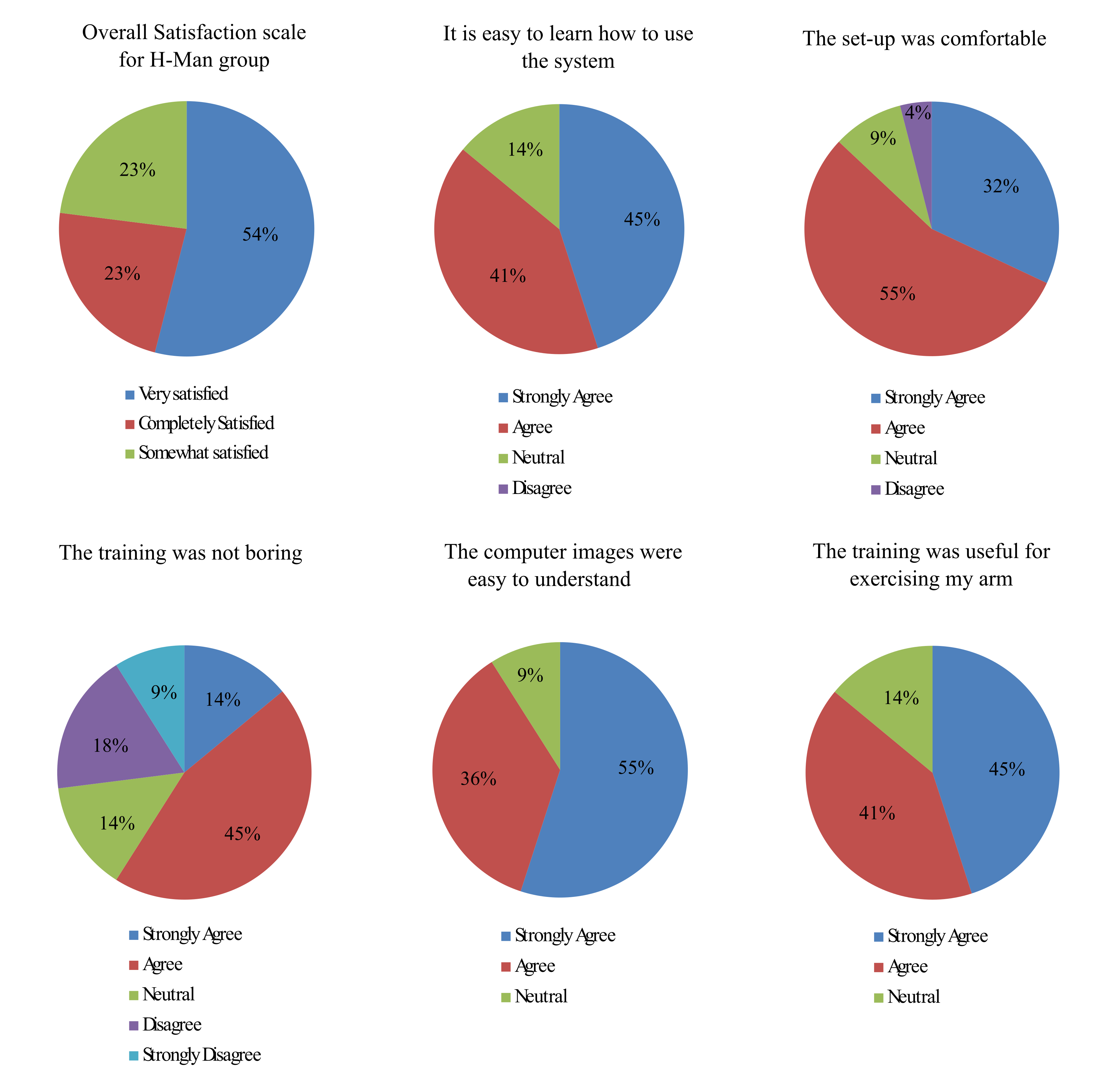


**Supplementary Figure 1.** Outcome measures on the experience, satisfaction and perceived benefits of robotic assisted therapy using H-Man recorded at the end of training (week 6; robotic group: n = 22).


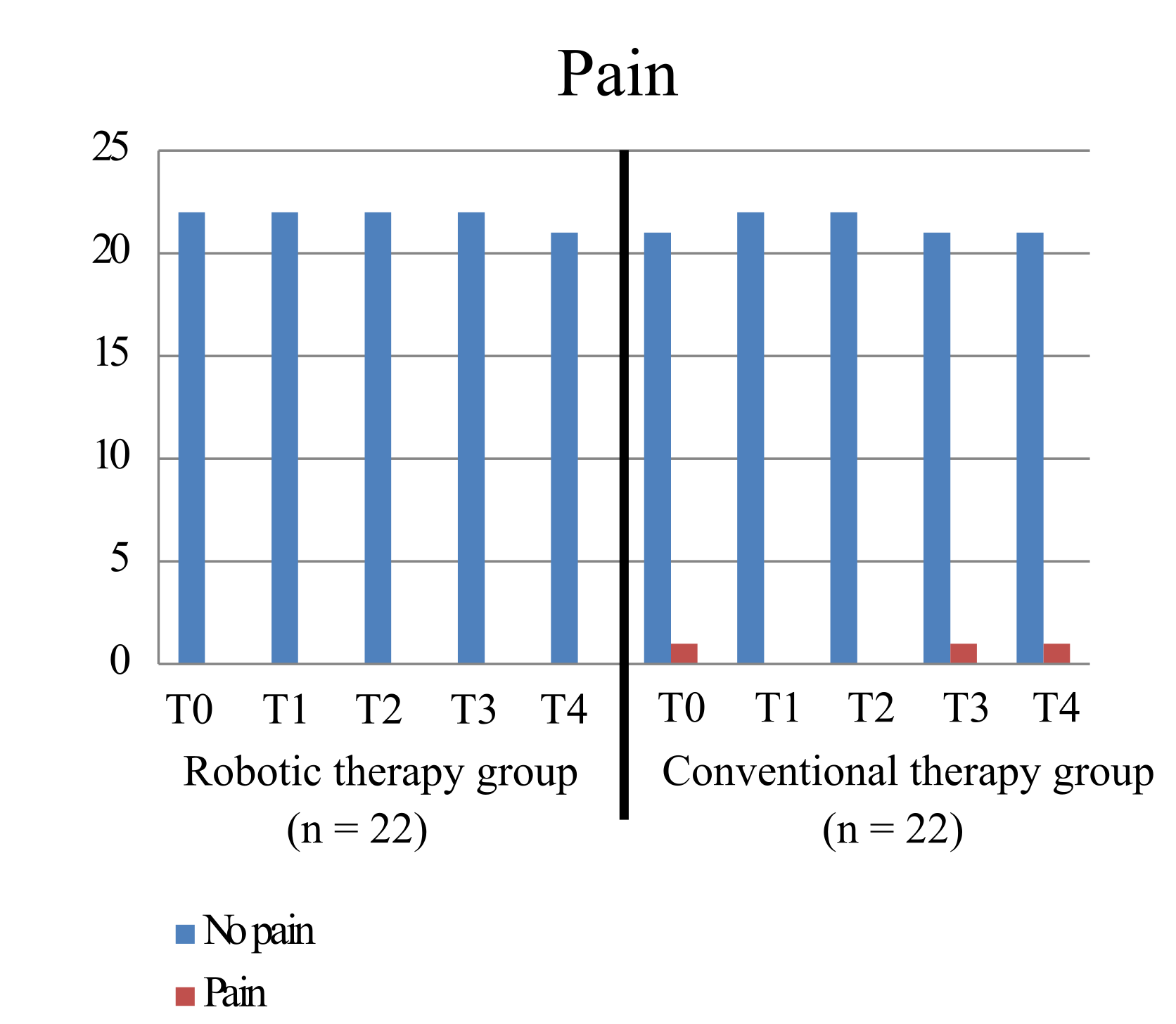


**Supplementary Figure 2.** Outcome measures on pain measured with the Modified Ashworth Scale (MAS) in the robotic (n = 22 for T0, T1, T2 and T3; n = 21 for T4) and conventional therapy (n = 22) groups recorded before (week 0: T0), during (week 3: T2 and week 6: T3) and after therapy (week 12: T4 and week 24: T5)


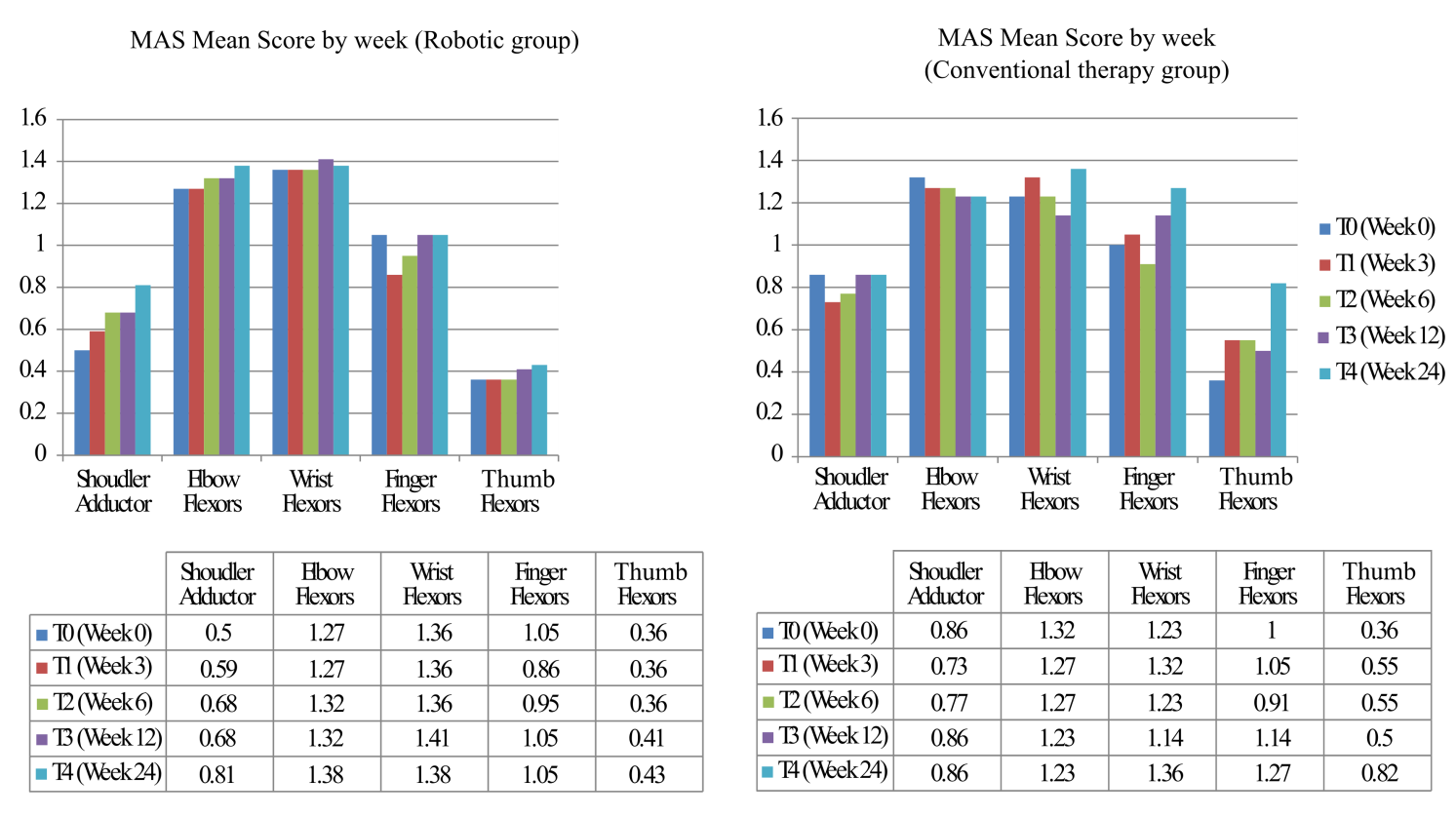


**Supplementary Figure 3.** Outcome measures in pain measured in MAS in the robotic and conventional therapy recorded before (week 0), during (week 3 and 6) and after therapy (week 12 and 24).

**
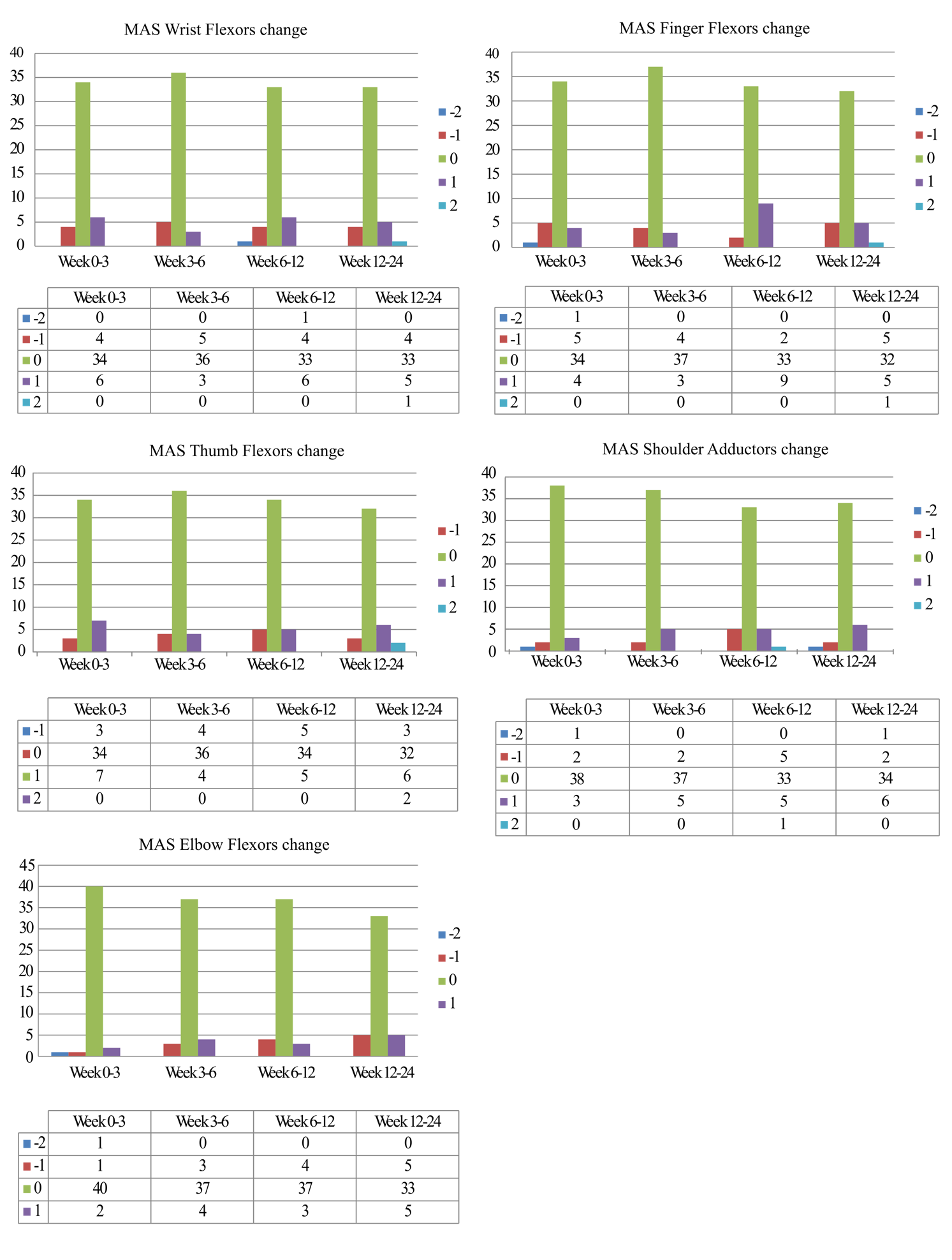
**

**Supplementary Figure 4.** Outcome measures on pain measured with MAS in all stroke participants (n = 44 for week 0, 3, 6 and 12; n = 43 for week 24)
